# Communicating Emotion: Vocal Expression of Linguistic and Emotional Prosody in Children With Mild to Profound Hearing Loss Compared With That of Normal Hearing Peers

**DOI:** 10.1097/AUD.0000000000001399

**Published:** 2023-06-15

**Authors:** Tjeerd J. de Jong, Marieke M. Hakkesteegt, Marc P. van der Schroeff, Jantien L. Vroegop

**Affiliations:** Department of Otorhinolaryngology and Head and Neck Surgery, University Medical Center Rotterdam, Rotterdam, the Netherlands

**Keywords:** Children, Cochlear implants, Emotional prosody, Hearing loss, Hearing aids

## Abstract

**Objectives:**

Emotional prosody is known to play an important role in social communication. Research has shown that children with cochlear implants (CCIs) may face challenges in their ability to express prosody, as their expressions may have less distinct acoustic contrasts and therefore may be judged less accurately. The prosody of children with milder degrees of hearing loss, wearing hearing aids, has sparsely been investigated. More understanding of the prosodic expression by children with hearing loss, hearing aid users in particular, could create more awareness among healthcare professionals and parents on limitations in social communication, which awareness may lead to more targeted rehabilitation. This study aimed to compare the prosodic expression potential of children wearing hearing aids (CHA) with that of CCIs and children with normal hearing (CNH).

**Design:**

In this prospective experimental study, utterances of pediatric hearing aid users, cochlear implant users, and CNH containing emotional expressions (happy, sad, and angry) were recorded during a reading task. Of the utterances, three acoustic properties were calculated: fundamental frequency (F0), variance in fundamental frequency (SD of F0), and intensity. Acoustic properties of the utterances were compared within subjects and between groups.

**Results:**

A total of 75 children were included (CHA: 26, CCI: 23, and CNH: 26). Participants were between 7 and 13 years of age. The 15 CCI with congenital hearing loss had received the cochlear implant at median age of 8 months. The acoustic patterns of emotions uttered by CHA were similar to those of CCI and CNH. Only in CCI, we found no difference in F0 variation between happiness and anger, although an intensity difference was present. In addition, CCI and CHA produced poorer happy–sad contrasts than did CNH.

**Conclusions:**

The findings of this study suggest that on a fundamental, acoustic level, both CHA and CCI have a prosodic expression potential that is almost on par with normal hearing peers. However, there were some minor limitations observed in the prosodic expression of these children, it is important to determine whether these differences are perceptible to listeners and could affect social communication. This study sets the groundwork for more research that will help us fully understand the implications of these findings and how they may affect the communication abilities of these children. With a clearer understanding of these factors, we can develop effective ways to help improve their communication skills.

## INTRODUCTION

In spoken language, both linguistic and paralinguistic information are essential to convey the content and the emotional context of a message (Van de Velde et al. 2019). Paralinguistic information is generally referred to as “prosody,” which can be described as information in speech that is not conveyed through syntax (the arrangement of words), but rather through phonetics, such as change in pitch and intensity (Murray et al. 1993), and temporal elements such as pace and rhythm. Prosody plays an important role in social interaction and communication as a whole (Bosacki et al. 2004; Wiefferink et al. 2012). Children use prosody in their speech to express emotion as early as the first year of life, and it develops further until the age of 13 (Banse & Scherer 1996; Scheiner et al. 2006; Flom et al. 2007; Peppe et al. 2007; Aguert et al. 2013).

As young listeners map distinct acoustical features of the voice onto speakers’ emotional states, they learn to decode emotional information conveyed by a speaker (Banse & Scherer 1996). Typically developing children reveal a wide range of abilities in this skill, with some children having more difficulty recognizing and categorizing emotions from voices than others. However, with extensive experience and learning, the vocal-emotional mapping yields an efficient auditory mechanism for rapidly ascertaining the emotional state of a communication partner (Morningstar et al. 2018).

Hearing loss can negatively impact the ability to detect prosodic cues, even with auditory rehabilitation (Moore 1996; Green et al. 2004; McDermott 2004; Wei et al. 2007; Chatterjee & Peng 2008; Most et al. 2009). Studies have shown that children with hearing loss have difficulties with pitch perception and accurately identifying prosody (Peng et al. 2008, 2017; Chin et al. 2012; Lin et al. 2021; Ren et al. 2021), which can limit their prosodic expression (Nakata et al. 2012; Wang et al. 2013; Chatterjee et al. 2019; Van de Velde et al. 2019). Caution is necessary when comparing results between languages. Languages with shared phonological features, such as English and Dutch—with a potential overlap of up to 90% (William Forde & Balkwill 2006), may have more similarities in conveying emotions than those with different roots.

Previous studies have analyzed children’s prosodic expression potential through assessment of various acoustic properties, including fundamental frequency, variations in fundamental frequency, and intensity. These properties represent crucial aspects of prosody and are relatively easy to measure and examine. Chatterjee et al. (2019) discovered that children with cochlear implants (CCI) had less varied prosodic expression in terms of fundamental frequency and its SD compared with normal hearing peers. Furthermore, other studies have indicated that subjective ratings of sadness were lower as expressed by CCI than those with normal hearing (Nakata et al. 2012; Wang et al. 2013; Van de Velde et al. 2019).

As most pediatric studies investigating emotional prosodic conveyance focused on children with severe to profound hearing loss using cochlear implants, very little is known about the prosodic expression by children with mild to severe hearing loss wearing hearing aids. These children with milder hearing losses may also experience significant speech recognition challenges because of reduced audibility and decreased temporal and spectral processing sensitivity and selectivity (Bronkhorst 2000). However, in comparison to CCI who have significantly poorer spectral resolution as the temporal fine structure cues are largely discarded, children with milder degrees of hearing loss may perform relatively better in prosodic conveyance. One study showed that the perception of emotions by children with a lower degree of hearing loss was not different from that of children with normal hearing (CNH; Cannon & Chatterjee 2019). By contrast, a different study, involving a mixed group of nine CHA and six with CCI, all with different degrees of hearing loss, found limitations in both prosodic perception and expression and degree of hearing loss was a predictor for the participants’ prosodic perception performance (Kalathottukaren et al. 2017). However, the investigators did not find a correlation between prosodic perception and expression. Given the conflicting results of these studies, it would be desirable to measure how effectively children wearing hearing aids (CHA) can express vocal emotion.

In the present study, we compared the prosodic expression potential of children with mild to severe hearing loss wearing hearing aids bilaterally or unilaterally to that of age-equivalent CCI and CNH. The following acoustic properties were established: fundamental frequency, variance in fundamental frequency, and mean intensity of the children’s utterances. We aimed to investigate (1) the difference in acoustic properties of emotional utterances of CHA compared with CCI and CNH, (2) the individual acoustic differences between the three emotions within the three groups, and (3) acoustic contrasts between emotions uttered by CHA or CCI compared with CNH, as these contrasts can provide insight into how emotions can be distinguished from each other. Acoustic contrasts have been used in previous studies and are an essential component in investigating the potential for prosodic expression.

Given the cochlear implants’ limitations in sound representation and the previous findings on their users’ prosodic conveyance, we hypothesized fewer acoustic differences and lower acoustic contrasts between emotions in this group than in the CNH. Although the impact of lower degrees of hearing loss on prosodic expression is not yet known, we expect this group to also experience limitations in prosodic expression compared with those with normal hearing.

## MATERIALS AND METHODS

### Participants

Three groups of participants were included in the study: CHA, CCI, and CNH. To ensure that all participants could complete the reading task without age-related reading difficulties, we set the inclusion age to begin at 7 years. For this study, we included children up to the age of 13, because it is commonly believed that prosodic expression would have leveled off beyond this age. Because we wanted to include children with milder losses, hearing thresholds had to be at least 35 dB HL on 0.5, 1, 2, and 4 kHz on average in the best hearing ear. To ascertain the inclusion of adequately fitted participants, intervention with hearing aid or cochlear implant had to be at least 6 months before inclusion. To ensure the inclusion of participants with an appropriate reading level. Parents were asked to provide information regarding their child’s reading performance and confirm that their child had reached or surpassed a third-grade reading level. Of the children who were included, all were able to complete the task sufficiently. All participants with hearing loss were being treated in the Audiology Department of the Erasmus University Medical Center for hearing aid fitting, cochlear implant programming, and regular annual appointments with audiologists and/or speech therapists who worked closely with the children and their families. The focus of these appointments was to optimize the performance of their hearing devices through proper adjustments, and providing support by a speech therapist. In addition, the rehabilitation process involved other assistance, such as providing assistive listening devices, to support effective communication in various settings. These interventions were tailored to individual needs, aiming to enhance participants’ hearing experience, optimize communication, and mitigate challenges associated with hearing loss.

The participants with cochlear implants used either a Cochlear (Nucleus 6, Nucleus 7, or Kanso I) or an Advanced Bionics (Naida Q70 or Naida Q90) device. CNH, between the ages of 6 and 13 years and without any history of hearing loss or current complaints of such, were recruited for the study through employees of the Erasmus University Medical Center and their personal networks. Ages did not differ between the groups with hearing loss (10.3 yr) and normal hearing (10.0 yr, *p* = 0.26). A total of 75 children participated in this study; the sample comprised 26 CHA, 23 CCIs, and 26 with CNHs (see Table 1 for participant demographics). All participants were native Dutch speakers. All caregivers signed an informed consent form before their child’s participation. The Medical Ethics Committee of the Erasmus University Medical Center Rotterdam, The Netherlands has reviewed the research protocol and has judged that the rules laid down in the Medical Research Involving Human Subjects Act do not apply to this research proposal. The study was conducted according to the principles of the Declaration of Helsinki (64th World Medical Association 2013) and the General Data Protection Regulation.

**TABLE 1.** Demographics and distribution of values across groups of hearing status

Characteristic	Children With Cochlear Implant (n = 23)	Children With Hearing Aid (n = 26)	Children With Normal Hearing (n = 26)	*p*
Age (yrs)				
Mean (SD)	9.9 (1.9)	10.7 (1.8)	10.0 (1.6)	0.92
Sex				
Male	13 (57%)	15 (58%)	13 (50%)	0.84
Female	10 (43%)	11 (42%)	13 (50%)	
Unaided pure-tone thresholds (PTA 0.5–4 kHz, better ear)				
Mild (35–40 dB HL)	—	5 (19%)	—	n/a
Moderate (41–60 dB HL)	—	12 (46%)	—	
Severe (61–80 dB HL)	—	8 (31%)	—	
Profound (>80 dB HL)	—	1 (4%)	—	
Speech perception in quiet (65 dB SPL, %)				
Mean (SD)	94.8 (5.8)	96.5 (5.6)	—	0.30
Onset of hearing loss				
Congenital	15 (65%)	15 (58%)	—	0.60
After birth	8 (35%)	11 (42%)	—	
Age at intervention (mean years [SD])			—	
Congenital onset of hearing loss	0.7 (0.7)	1.8 (2.0)	—	0.08
After birth onset of hearing loss	5.7 (3.2)	7.3 (3.4)	—	0.32
Device experience (mean years [SD])	7.4 (3.4)	6.6 (3.7)	—	0.52
Hearing device				
Bilateral	15 (65%)	23 (88%)	—	0.05
Unilateral	1 (5%)	3 (12%)	—	
Bimodal	7 (30%)	—	—	
Mode of communication				
Oral	23 (100%)	25 (96%)	26 (100%)	0.34
Combined oral and sign language		1 (4%)		

published online ahead of print June 15, 2023.

Table displays the demographics and audiological characteristics of all participants, divided in groups of hearing status. Results were compared between groups with the analysis of variance. Device experience was calculated as the time between device intervention and participation. A hyphen-minus indicates that the variable is not available in that specific group.

PTA, pure-tone average.

### Test Procedure

The prosodic expression of the children was tested subsequent to the children’s scheduled outpatient visit. Testing took place between January 2021 and June 2021. Due to the COVID-19 pandemic, not all CNH group were able to visit the hospital; therefore, children were tested either at the hospital (n = 13) or at home (n = 13). No differences in ambient noise intensity were found between recordings made at home (average 31.6 dB SPL) or at the hospital (average 29.9 dB SPL; Mann–Whitney U = 241, n = 52, *p* = 0.27). The same test protocol and set-up were used throughout the study, both in the hospital and at home. No differences in mean fundamental frequency (F0), variance in F0, and intensity, for all three emotions were observed between home and hospital recordings. All testing was performed in one session, taking between 2 and 5 minutes. Tests were performed in a quiet room, under the supervision of an investigator who was aware of the purpose of the study. Tasks were explained in live voice.

To test the children’s prosodic expression, they were asked to read aloud 18 sentences (see Table 2). The sentences were chosen to be easily readable for children with at least third-grade reading levels and to be of neutral content, allowing the focus of the test to be solely on the children’s prosodic expression.

**TABLE 2.** Sentences that participants were instructed to read during the task

Part	No.	Dutch Sentence	English Translation	Expressed Emotion
Practice	1	Morgen komt er bezoek.	There will be visitors tomorrow.	Happy, sad, angry
	2	Een groene bal.	A green ball.	Happy, sad, angry
Recording	3	De bal gaat in het doel.	The ball goes into the goal.	Happy, sad, angry
	4	De hond loopt op straat.	The dog is walking on the street.	Happy, sad, angry
	5	Ik zie de juf.	I see the teacher.	Happy, sad, angry
	6	Dit is van mij.	This is mine.	Happy, sad, angry

Sentences are grouped per part of the task. The English translation and expressed emotion are denoted behind each sentence. Sentences 1 and 2 were read in a fixed order, that alternated between sentence and emotions. Sentences 3–6 were read in a random order.

**Fig. 1.**
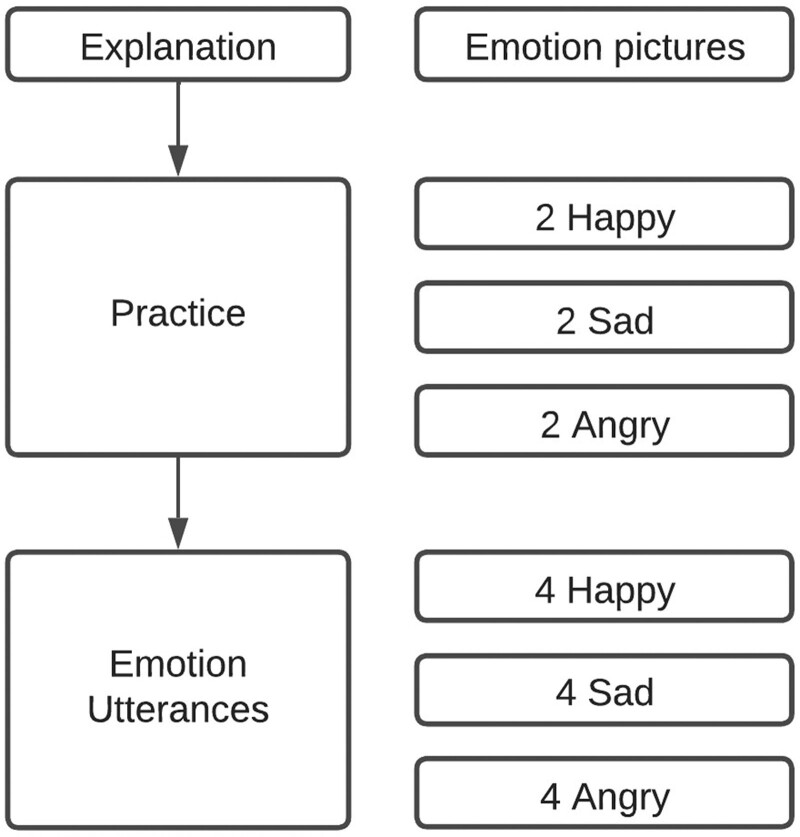
The three parts of the task. In the left column, the parts are denoted. In the right column, the type of sentences are denoted, with their relative emotions.

**Fig. 2.**
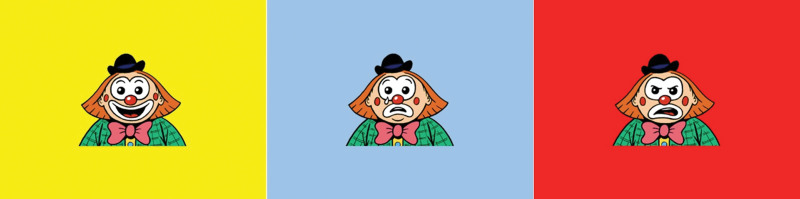
The three emotions, happy, sad, and angry, depicted by the three clowns with corresponding facial expressions, which were used. The illustrations on the sheets were made by Jop Luberti. These illustrations are published under the CC BY NC 4.0 license. (https://creativecommons.org/licenses/by-nc/4.0/).

In the first part of the reading task (see Fig. 1 for an overview) three pictures of a clown were shown, each with a different facial expression (happy, sad, angry; see Fig. 2). These pictures have previously been used in another prosody study, and were found to be suitable for accurately depicting emotions for a children’s audience (Nagels et al. 2020). Participants were asked if they could recognize the facial expressions. The test would proceed if the participant named the three emotions correctly. Children were instructed to continue reading the presented sentences aloud, now while vocally expressing the emotion of the clown depicted below the sentence. The second part contained two practice sentences that children were instructed to read once per emotion. The third part contained four sentences, threefold, with either a happy, sad, or angry clown, in a randomized order (the sentences and practice sentences can be found in Table 2). During each round, only neutral supportive feedback was given by the investigator.

During the task, speech was transduced using an AKG MicroMic C544L microphone attached to a headset, placed 2–3 cm from the participant’s mouth. The signal was converted with a Scarlett Solo second-generation 2-in/2-out USB preamplifier, and recorded with a Dell laptop with an Intel i5-6200 U central processing unit with a sampling frequency of 44.1 kHz.

To provide a comprehensive overview of the participants’ speech perception abilities, we have included their scores in the demographics. Aided speech perception in quiet was measured during clinical follow-up of the hearing loss and these data were retrieved from the patient files. It was measured with the Dutch speech test of the Dutch Society of Audiology (Bosman and Smoorenburg 1995). Word lists were presented at 65 dB SPL, and a Decos audiology workstation, version 210.2.6 was used.

### Acoustic Analyses

Recordings were analyzed with Praat phonetic analysis software version 6.1.40 (Phonetic Sciences, University of Amsterdam; Boersma 2001). For the acoustic analyses, a script was designed to systematically compute the acoustic properties of the utterances. For general applicability of the results, acoustic properties of individual utterances were examined similarly to those in previous literature (Chatterjee et al. 2019): mean F0 (in Hz), the variation in fundamental frequency (SD of F0; in Hz), and the mean intensity (in dB). Measures were calculated on voiced periods only, thereby disregarding silence or pause by the participants. Onset and offset of the utterances were estimated by one author (T.J.d.J.) using objective criteria. The criteria were (1) onset of a sentence is marked by the first rise or fall in the oscillogram within 0.05 seconds before the utterance, (2) offset of a sentence is marked by the last rise or fall in the oscillogram before silence, or (3) in case that the onset or offset are obscured by additive noise (e.g., breathing or movement), onset and offset are marked by the strongest increase and respectively decrease in signal on the spectrogram. Onset and offset estimations were checked for accuracy by an independent researcher through random sampling. The variation in fundamental frequency was calculated using the default pitch range (75–500 Hz).

### Sample Size Calculation

An a priori power analysis was performed, with a required power of 0.8 and an alpha-error level of 0.05. We based the power analysis on the findings of Wang et al. (2013). They described a difference in F0 of 25 Hz in happy utterances between CCI and CNH reference group (with a mean F0 of 275 HZ versus 275 Hz, respectively; Wang et al. 2013). Accordingly, an F0 difference of 25 Hz between the CCI and the CNH group was expected. Still, a smaller difference was expected between the CCI and CHA group. Therefore, a smaller effect size of 10 Hz was used for sample size calculations. Wang et al. (2013) provided SDs of 12 and 14. With these data, we calculated an intended sample size of 24 children per group.

### Data Analysis

We compared the distribution of demographic variables between the three groups using independent samples t tests for normally distributed data and nonparametric tests for non-normally distributed data.

Distribution of acoustic properties was investigated for normality with the Kolmogorov-Smirnov test (Kolmogorov 1933). This test indicated that the data on acoustic properties was normally distributed for sad utterances. Happy and angry utterances had non-normally distributed data on F0 variation, mean intensity, and mean F0, respectively. Therefore, we used nonparametric tests in all analyses.

First, group comparisons (CCI versus CHA versus CNH) were made of the averages in mean F0, F0 variation, and mean intensity per emotion (happy, sad, and angry). These comparisons were performed using Kruskal-Wallis tests for independent samples.

In the second analysis, the averages in mean F0, F0 variation, and mean intensity were compared between emotions (i.e., happy versus sad versus angry) within the three different groups, with a Wilcoxon Signed-Ranks test.

In the third analysis, emotion contrasts were investigated. The contrasts were calculated as the ratio between the acoustics of two emotions per utterance, per child. For instance, mean F0 of sentence 11 expressed with happy intent by one participant, was divided by the mean F0 of sentence 11 expressed with sad intent by the same participant. Because decibels, the unit for intensity, expresses a ratio itself, we used the absolute difference in dB |dB_x_–dB_y_| as emotion contrast for intensity. This approach was similar to that of Chatterjee et al. (2019). The average of ratios was calculated per emotion contrast. This resulted in three emotion contrasts (happy versus sad, happy versus angry, sad versus angry) per acoustic property per child. The group averages of the three emotion contrasts per acoustic property were compared between groups with a Kruskal-Wallis test.

Displayed *p* values are corrected for family-wise error, for which the Benjamini–Hochberg procedure was followed in all analyses (Benjamini and Hochberg 1995). An alpha level of 0.05 was set as the threshold for significance. All statistical analyses were performed in IBM SPSS Statistics 25.0.0.1.

## RESULTS

### Participants

Data were collected from a total of 75 Dutch-speaking children. Ages ranged from 7.2 to 12.9 years (M ± SD = 10.2 ± 1.8). For CHA, pure-tone thresholds were mostly moderate (n = 12; 46%), 5 children (19%) had mild, and 9 (35%) had severe-profound hearing losses. Children in this group had an average device experience at moment of testing of 6.6 years, and 23 children (88%) wore hearing aids bilaterally. In the CCI group, children had a mean device experience of 7.4 years, used cochlear implants bilaterally in 15 of the cases (65%), and 7 (30%) were bimodal users (demographics are displayed in Table 1). The distribution of age and sex was similar in all three groups. Between CCI and CHA, there were no differences in aided speech perception in quiet, onset of hearing loss, age at intervention, device experience, or unilateral/bilateral use of hearing devices.

### Group Differences

**Fig. 3.**
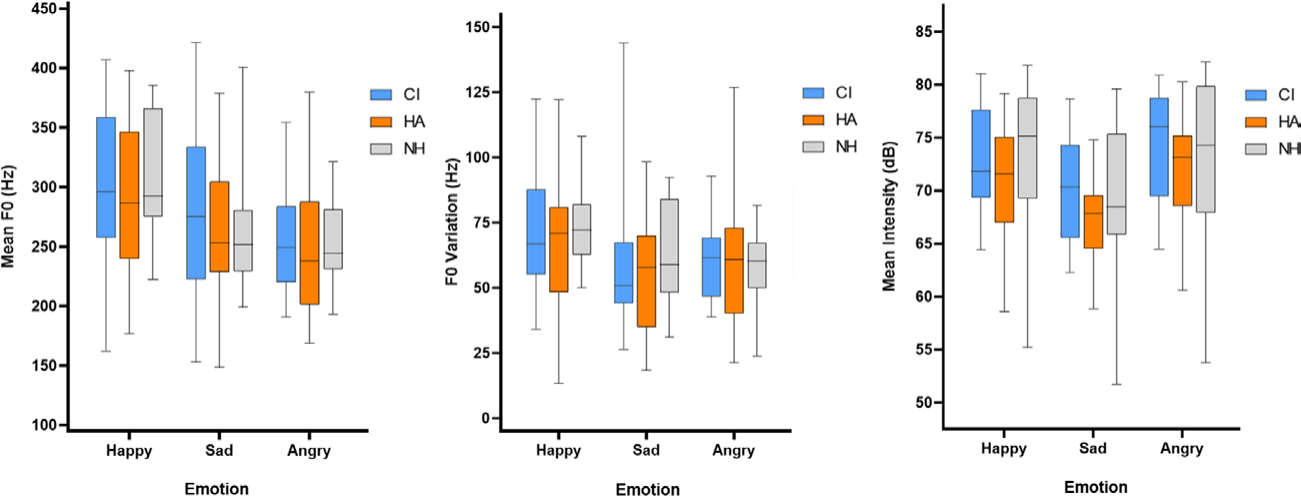
Distribution of acoustic properties across groups of hearing status. A, Distribution of mean F0 per group of hearing status by category of emotion. B, Distribution of F0 variation per group of hearing status by category of emotion. C, Distribution of mean intensity per group of hearing status by category of emotion. This figure contains boxplots of the acoustic properties of the utterances. Boxes and whiskers each represent a 25% share of the distribution of the data. From top to bottom, the figures, respectively, indicate the mean values of mean F0, F0 variation, and mean intensity across the utterances. F0 indicates fundamental frequency.

When regarding all children, we found no difference in mean F0, F0 variation, and mean intensity distribution between the three groups for any of the emotions (see Table 3 for the acoustic profiles). This implies that the utterances produced by CHA and CCI are similar to those produced by CNH in mean F0, F0 variation, and mean intensity (Fig. 3A–C displays the distribution of acoustic properties across groups).

**TABLE 3.** Acoustic profiles of three emotions: means and SDs of acoustic parameters

Emotion	Acoustic Variable	Group			*p*
		CHA	CCI	CNH	
Happiness	Mean F0 (Hz)	292	301	309	0.59
	SD	66	68	46	
	F0 variation (Hz)	66	71	74	0.58
	SD	24	23	15	
	Intensity (dB)	71	73	73	0.80
	SD	5	5	7	
Sadness	Mean F0 (Hz)	266	278	260	0.38
	SD	63	67	43	
	F0 variation (Hz)	56	58	64	0.42
	SD	23	24	19	
	Intensity (dB)	67	70	69	0.84
	SD	4	5	7	
Anger	Mean F0 (Hz)	249	258	254	0.35
	SD	57	46	34	
	F0 variation (Hz)	58	61	59	0.34
	SD	22	16	14	
	Intensity (dB)	72	75	73	0.40
	SD	5	5	8	

Table displays the means per group.

CCI, children with cochlear implants; CHA, children wearing hearing aids; CNH, children with normal hearing; F0, fundamental frequency.

### Acoustic Differences Between Categories of Emotional Intent

**Fig. 4.**
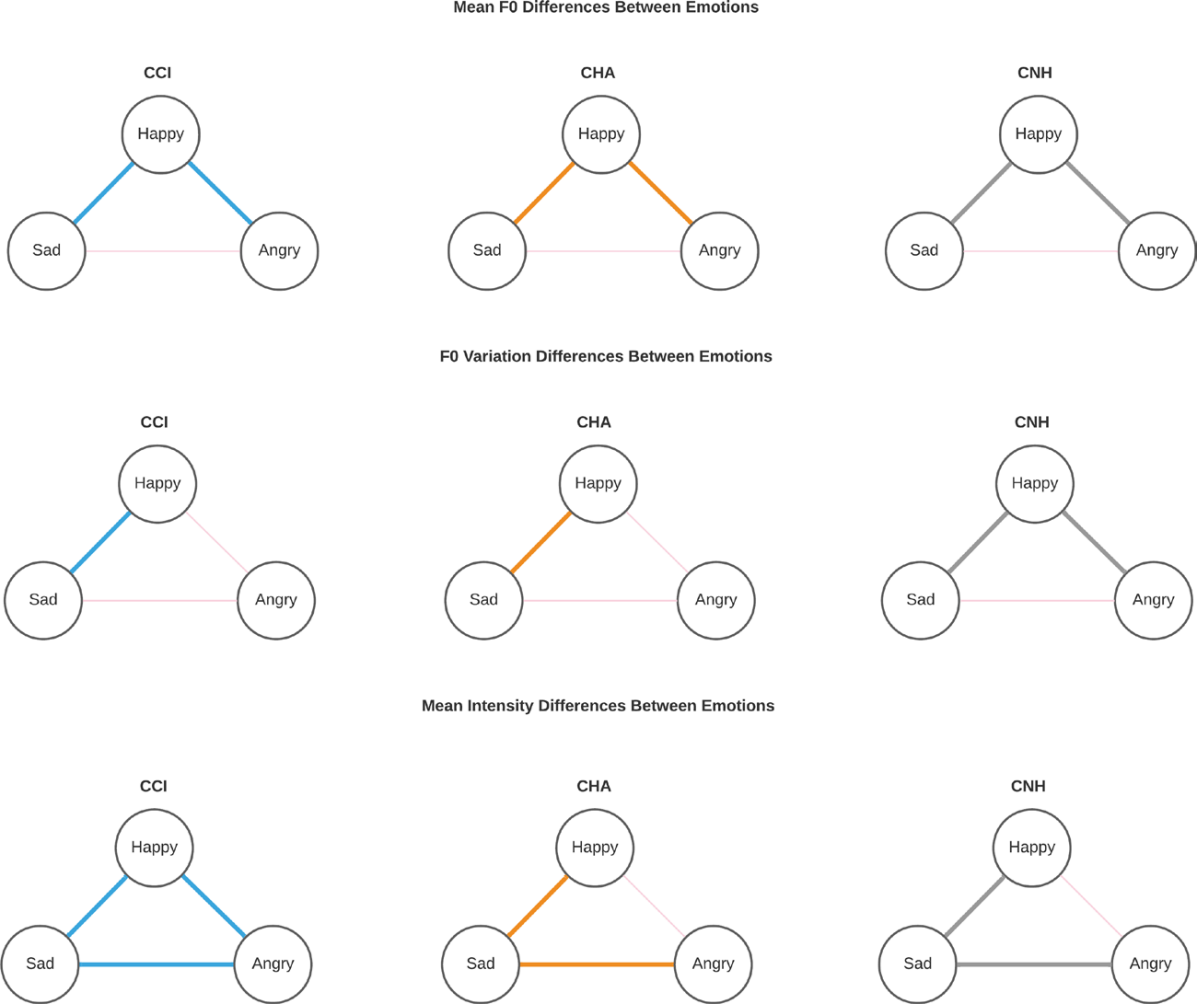
Acoustic differences between emotions per group of hearing status. This figure illustrates the differences in mean acoustic properties of utterances produced by children with cochlear implants (CCI), children with hearing aids (CHA), or children with normal hearing (CNH) during the task. The individual categories of emotions are denoted in the circles. Happy intent was compared with sad and angry, and sad was compared with angry intent. Significant differences are indicated by the thick colored lines: blue for CCI, orange for CHA, and gray for CNH.

Within each group, the differences between emotions were compared (the comparisons are illustrated in Fig. 4, and test statistics for the individual comparisons are given in Supplemental Appendix 1, Supplement Digital Content, http://links.lww.com/EANDH/B183).

#### Differences in Mean F0 Between Emotions

In all groups, the average of mean F0 was significantly higher in happiness compared to sadness (CHA: *p* < 0.01, CCI: *p* = 0.02, CNH: *p* < 0.01) and anger (CHA *p* < 0.01, CCI: *p* < 0.01, CNH: *p* < 0.01; see also Supplemental Appendix 1, Supplement Digital Content, http://links.lww.com/EANDH/B183 and Fig. 3A). Mean F0 was not different between sad and angry utterances in all groups.

#### Differences in F0 Variation Between Emotions

F0 variation was significantly lower in sadness versus happiness in all groups (CHA: *p* = 0.03, CCI: *p* < 0.01, CNH: *p* = 0.01), whereas happiness only had a higher F0 variation than anger in CNH (*p* < 0.01). In CHA and CCI, no difference in F0 variation could be found between happiness and anger.

#### Differences in Mean Intensity Between Emotions

Intensity differences between happiness and sadness (and sadness and anger) were significant for all groups. Group angry utterances were louder than happy, which in turn were louder than sad utterances. On average, mean intensity differed significantly between all three emotions in CCI. This was the only group that exhibited significant intensity differences between happiness and anger, in contrast with both CNH and CHA.

#### Emotion Contrasts

We compared the distribution of emotion contrasts between the three groups. Between CCI, CHA, and CNH, there was no difference in emotion contrasts, except for the emotion contrast between happy and sad utterances. For mean F0, the happy–sad contrasts in CCI and CHA were significantly poorer than those in CNH (*H*(2) = −14, *p* = 0.02 for CCI versus CNH, *H*(2) = −13, *p* = 0.04 for CHA versus CNH). Test statistics for all emotion contrasts are provided in Supplemental Appendix 2, Supplement Digital Content, http://links.lww.com/EANDH/B183.

Post hoc analysis showed no correlation between the size of emotion contrasts and age at testing and speech perception performance in quiet. Sad–angry contrasts in both mean F0 (ρ(47) = −0.39, *p* = 0.04) and variance in F0 (ρ(47) = −0.42, *p* = 0.04) correlated significantly with device experience, in terms that children with lower device experience had greater acoustic contrasts between sadness and anger. Device experience was significantly higher in children with a congenital onset of hearing loss (9.1 years versus 4.0 years later onset; Mann–Whitney U = 44, n = 46, *p* < 0.01).

## DISCUSSION

In this prospective study, we included 26 children with mild to severe hearing loss wearing hearing aids (CHA), and compared their prosodic expression with that of 23 CCI, and 26 CNH. On average, the distributions of mean F0, F0 variation, and mean intensity per emotion in CHA were similar to those in the CCI and CNH groups. CCI may well experience more difficulties with the perception of subtle variations in voice pitch due to a lack of resolved harmonics in cochlear implants and a lack of temporal fine structure coding—most current implants convey only temporal envelope information and discard the temporal fine structure. This could be a reason for the more monotonous expression of emotion in these children compared to that of CNH, a finding also reported by Chatterjee et al. (2019).

Chatterjee et al. (2019) described that CCI and CNH expressed happiness similarly. With sad expressions; however, they found a significantly higher mean F0 in CCI versus CNH. Acoustic investigation of angry expressions was performed neither in that study, nor in any other existing literature on prosodic acoustics (Nakata et al. 2012; Wang et al. 2013). In our study, CCI and CHA, on average, conveyed anger with a similar acoustic pattern as CNH. A remarkable finding, however, was that both CHA and CCI did not exhibit significant F0 variation differences between happiness and anger, in contrast with CNH. Contrarily, an intensity difference between these emotions was present in CCI, and a similar tendency was observed in CHA. It is therefore likely to assume that CCI, and at least a portion of the CHA, made more use of intensity than of F0 variation when expressing anger. Children with hearing loss possibly compensate for their lack in F0 variation with the use of intensity instead. This possible exchange in acoustic cues has been delineated by previous studies on voice emotion perception, where the removal of intensity cues resulted in poorer emotion perception scores by cochlear implants users (Xin et al. 2007; Peng et al. 2009, 2017; Winn et al. 2012). Until now, the possible exchange in acoustic features has not been identified in voice emotion expression studies. In the context of language acquisition, it is generally accepted that children’s ability to vocalize words accurately is closely linked to their ability to perceive and register spoken language (Goldin-Meadow 2015). Our results suggest that CCI follow a similar pattern of learning for prosody as for language. Specifically, we found that the way different acoustic cues interact with each other plays an important role in conveying emotions.

We used the ratio of acoustic values between emotions to investigate the emotion contrasts between groups. We found a poorer mean F0 contrast between happiness and sadness in CHA and CCI compared with that of CNH. Despite diminished perception of sound of even the CHAs and CCIs, these children were capable of expressing emotions distinguishable by acoustic patterns. This may be due to the auditory and linguistic rehabilitation that the children with hearing loss received, in accordance with Dutch national guidelines (Brienesse et al. 2013; CI-ON 2013), and early intervention as a result from the Dutch national newborn hearing screening. With adequate rehabilitation, children with hearing loss are able to reach age-equivalent language proficiency, including the use of pragmatic language (Yoshinaga-Itano et al. 2010; Walker et al. 2015; Dettman et al. 2016). Post hoc analyses revealed that children who experienced hearing loss at a later stage in life had higher levels of acoustic contrast between sadness and anger. One possible explanation for this finding is that these children had more exposure to sound during early childhood, which may have facilitated the development of their prosodic expression. This development may persist even after the onset of hearing loss. This stresses how important the early years are for both brain and linguistic development. Also exposure to speech and prosody in particular is very important. The positive outcomes seen in a study on prosodic rehabilitation programs further highlight the potential for effective interventions to improve prosodic parameters in children with hearing loss (Sobhy et al. 2022). Although hearing loss can pose challenges to emotional communication, these findings offer hope for rehabilitation and underscore the critical role of auditory intervention in supporting children’s linguistic and emotional development.

### Strengths and Limitations

The present study has several strengths: (1) it is the first to investigate emotional prosodic expression in children with mild to moderate hearing loss, contributing new information to the general understanding of prosodic conveyance in children with hearing loss, (2) it has a sample size exceeding that of previous investigations (Nakata et al. 2012; Wang et al. 2013; Kalathottukaren et al. 2017; Chatterjee et al. 2019; Van de Velde et al. 2019), resulting in increased statistical power, (3) it contains read aloud sentences instead of imitated utterances, giving way for the children’s own prosodic processing abilities. Nevertheless, several limitations do exist. First, although we analyzed the three most commonly investigated acoustic properties, prosody comprises more assets, such as rhythm and pace, to state emphasis (Banse & Scherer 1996). Analyzing these elements may enable to establish more subtle differences in prosody between children with hearing loss and CNH. Second, we used simulated emotions, rather than spontaneous expressions. The effects that we found may, therefore, deviate from daily practice. Children’s prosodic expression potential in everyday life may best be assessed by a group of (subjective) raters, similar to those in the children’s environment. Third, a wide range of degree of hearing loss was included. Likely, the greater the severity of hearing loss, the larger the impact on emotional prosodic expression. Future studies should include more pediatric hearing aid users to be able to compare results between children with different degrees of hearing loss. Fourth, the CNH did not perform an audiometry test; slight hearing losses may therefore not have been ruled out. Lastly, the selection of children that had at least third-grade reading level, could have introduced bias.

## CONCLUSIONS

The findings of this study suggest that on a fundamental, acoustic level, both CHA and CCI have a prosodic expression potential that is almost on par with normal hearing peers. However, there were some minor limitations observed in the prosodic expression of these children, it is important to determine whether these differences are perceptible to listeners and could affect social communication. This study sets the groundwork for more research that will help us fully understand the implications of these findings and how they may affect the communication abilities of these children. With a clearer understanding of these factors, we can develop effective ways to help improve their communication skills.

## ACKNOWLEDGMENTS

M.M.H. and J.L.V. were involved in conceptualization; T.J.d.J. was involved in data curation; T.J.d.J. was involved in formal analysis; M.P.S. and J.L.V. were involved in funding acquisition; T.J.d.J., M.M.H., M.P.S., and J.L.V. were involved in investigation; T.J.d.J., M.M.H., M.P.S., and J.L.V. were involved in methodology; T.J.d.J., M.M.H., M.P.S., and J.L.V. were involved in project administration; T.J.d.J., M.M.H., M.P.S., and J.L.V. were involved in resources; M.P.S. and J.L.V. were involved in software and supervision; M.M.H. and J.L.V. were involved in validation; T.J.d.J. was involved in visualization; T.J.d.J., M.M.H., M.P.S., and J.L.V. were involved in roles/writing—original draft; T.J.d.J., M.M.H., M.P.S., and J.L.V were involved in writing—review and editing.

## Supplementary Material


