# Supplementary material for: Communicating Emotion: Vocal Expression of Linguistic and Emotional Prosody in Children With Mild to Profound Hearing Loss Compared With That of Normal Hearing Peers
Source: Ear Hear. 2023 Jun 15;45(1):72–80. doi: 10.1097/AUD.0000000000001399 (PMC10718210; doi:10.1097/AUD.0000000000001399)
Supplement: Supplementary file 1 [file aud-45-072-s001.pdf]

Appendix 1. Test statistics for the individual acoustic differences between categories of emotional intent

Difference per group	Acoustic property	Test statistic	Z score	P value
Neutral vs. question				
CHA	Mean F0	11.7	-2.7	**< 0.01***
	F0 variation	6.2	-3.5	**< 0.01***
	Mean intensity	14.0	-3.7	**< 0.01***
CCI	Mean F0	5.5	-3.8	**< 0.01***
	F0 variation	2.0	-3.9	**< 0.01***
	Mean intensity	13.2	-3.6	**< 0.01***
CNH	Mean F0	6.4	-3.3	**< 0.01***
	F0 variation	13.3	-3.4	**< 0.01***
	Mean intensity	14.7	-4.1	**< 0.01***
Happy vs. sad				
CHA	Mean F0	10.0	-3.2	**< 0.01***
	F0 variation	9.8	-2.2	**0.03***
	Mean intensity	4.0	-4.2	**< 0.01***
CCI	Mean F0	8.6	-2.4	**0.02***
	F0 variation	9.4	-2.8	**< 0.01***
	Mean intensity	2.0	-4.1	**< 0.01***
CNH	Mean F0	3.3	-4.2	**< 0.01***
	F0 variation	8.6	-2.5	**0.01***
	Mean intensity	0.0	-4.5	**< 0.01***
Happy vs. angry				
CHA	Mean F0	10.7	-3.6	**< 0.01***
	F0 variation	13.7	-2.0	0.06
	Mean intensity	14.7	-1.9	0.07
CCI	Mean F0	6.3	-3.0	**< 0.01***
	F0 variation	9.1	-1.4	0.15
	Mean intensity	13.9	-2.6	**< 0.01***
CNH	Mean F0	2.0	-4.4	**< 0.01***
	F0 variation	6.0	-4.0	**< 0.01***
	Mean intensity	11.9	-0.2	0.82
Sad vs. angry				

CHA	Mean F0	11.2	-1.9	0.07
	F0 variation	12.5	-0.3	0.75
	Mean intensity	14.0	-4.4	**< 0.01***
CCI	Mean F0	11.1	-1.5	0.14
	F0 variation	12.2	-1.4	0.17
	Mean intensity	12.0	-4.2	**< 0.01***
CNH	Mean F0	13.4	-1.1	0.32
	F0 variation	10.5	-1.5	0.15
	Mean intensity	14.5	-4.4	**< 0.01***

Notes: In this table, test statistics, Z scores and p values are given for the Wilcoxon Signed Ranks test used in the analysis of acoustic differences between emotions within groups. Significance is indicated with an asterisk and bold print. CHA is an abbreviation for children with hearing aids; CCI: children with cochlear implants; CNH: children with normal hearing; F0: fundamental pitch.

Appendix 2. Test statistics for the emotion contrasts between groups

Contrast	Acoustic property	Kruskal-Wallis H	P value
Neutral vs. question			
	Mean F0	3.4	0.19
	F0 variance	1.6	0.44
	Mean intensity	3.11	0.21
Happy vs. sad			
	Mean F0	6.6	**0.04***
	F0 variance	1.7	0.42
	Mean intensity	3.4	0.18
Happy vs. angry			
	Mean F0	1.1	0.58
	F0 variance	2.8	0.25
	Mean intensity	5.0	0.08
Sad vs. angry			
	Mean F0	0.44	0.80
	F0 variance	5.0	0.08
	Mean intensity	1.8	0.42

Notes: In this table, test statistics and p values are given for the Kruskal-Wallis test used in the analysis of emotion contrasts per acoustic property

between groups. The third and fourth column display the variance in emotion contrasts between CHA, CCI and CNH. Significance is indicated with an asterisk and bold print. CHA is an abbreviation for children with hearing aids; CCI: children with cochlear implants; CNH: children with normal hearing; F0: fundamental pitch.
